# Enhancing Understanding and Acceptance of Equipment Localization: Mixed Methods Study With Clinic Staff and Potential Patients

**DOI:** 10.2196/79583

**Published:** 2026-04-27

**Authors:** Angela Fiedler, Sabine Patzl, Melissa Schütz, Astrid Schütz

**Affiliations:** 1 Department of Psychology, University of Bamberg, Markusplatz 3, Bamberg, 96047, Germany, +49(0)951/863-1871

**Keywords:** digital health, informed consent, health care staff, understanding, implementation, patient privacy, real-time location systems

## Abstract

**Background:**

Digital technologies, such as equipment localization systems, can help clinics use mobile devices more efficiently. Their successful implementation, however, depends not only on technical feasibility but also on how staff and patients perceive and understand these systems.

**Objective:**

This research used 2 complementary studies to (1) obtain an initial picture of clinic staff attitudes toward the localization of vacuum-assisted closure (VAC) pumps and related concerns and (2) examine whether a simple layout change in a privacy policy (using guiding questions vs standard text) is associated with greater subjective understanding and acceptance among potential patients.

**Methods:**

In study 1, 38 employees of a German clinic completed a short survey assessing their comfort with and perceived usefulness of VAC pump localization and answered an open-ended question about reservations or concerns. Quantitative responses were analyzed descriptively, and free-text answers were coded using qualitative content analysis. In study 2, 498 participants from an online sample took part in a preregistered experiment. They were randomly assigned to read either a standard privacy policy information sheet or an otherwise identical version supplemented with guiding questions. Subjective understanding of the information and acceptance of the policy were then assessed and analyzed using rank-based regression models controlling for sociodemographic covariates.

**Results:**

Clinic staff in study 1 generally reported high levels of comfort (mean 7.34, SD 2.75) and perceived usefulness (mean 7.29, SD 2.69) regarding localization on 0‐10 scales. Concerns centered mainly on implementation feasibility, technical reliability, costs, and possible additional workload, rather than on privacy. In study 2, subjective understanding was slightly higher in the guiding-question layout condition than in the standard layout condition (mean 3.37, SD 0.63 [n=248] vs mean 3.24, SD 0.68 [n=250]); this difference was also significant in the rank-based regression model (b=0.13, SE=0.05, *t*=2.57; *P*=.01), and better understanding was associated with higher acceptance of the policy, explaining about 13.8% of the variance in acceptance scores.

**Conclusions:**

The exploratory findings suggest that, in the context of VAC pump localization, clinic staff generally view equipment tracking positively while still raising practical concerns that should be addressed during implementation. For potential patients, relatively small changes in the layout of privacy information—such as adding guiding questions—may support subjective understanding and willingness to accept data collection.

## Introduction

In large clinical settings, medical equipment must remain mobile to ensure that resources are available at the right place and time. However, locating mobile assets often requires time-consuming coordination and manual documentation, resulting in inefficiencies. Digital solutions—particularly real-time location systems (RTLSs), which are local infrastructures that identify and track the position of tagged objects or people via beacons distributed throughout the location of interest—offer promising avenues for improving workflows and resource allocation by providing real-time data on equipment use and availability [1-3].

One clinical example in which location-based data collection may be especially valuable involves vacuum-assisted closure (VAC) pumps, which are widely used in wound therapy. Although VAC therapy is well established, the manual process of tracking, cleaning, and redistributing pumps poses logistical challenges. When the stock is low, staff often need to call other units or request short-term rentals. Real-time localization could support more efficient allocation by enabling automated documentation, identifying device availability, and reducing cost as well as administrative burden [4].

Although the potential benefits of RTLSs include improved workflow, increased safety, and reduced cost [5-7], their successful implementation depends on more than technical infrastructure. On the one hand, health care workers are more likely to adopt new technologies if the benefits are clearly understood and they are actively involved in the implementation process [8]. Conversely, concerns about increased workload, privacy, and surveillance may hinder adoption [9,10].

On the other hand, patients’ privacy concerns will be more salient if location data can be linked to individuals. Thus, from a patient perspective, systems that track movement raise critical questions about data security, consent, and control over personal information [11]. Beyond data protection in a narrow sense, RTLS infrastructures can also enable fine-grained monitoring of individuals’ movements, which has raised broader concerns about surveillance, autonomy, and potential changes in workplace and care relationships in hospital and long-term care settings [9-11].

Although privacy policies and consent forms are designed to soothe privacy concerns and enable informed decisions, studies have consistently found that these documents are often too complex and that patients struggle to comprehend them—especially when they are presented in dense, text-heavy formats [12,13]. Health communication research has indicated that the layout of consent materials can significantly affect comprehension. Structured documents with headings, bullet points, and guiding questions have been shown to improve readability and facilitate understanding [14,15]. Because document layout directly shapes user trust, informed decision-making, and willingness to give consent, thoughtful designs are pivotal for digital solutions (such as RTLSs) in health contexts.

This research investigated the perspectives of both clinic staff and patients on location-based data collection in health care, using the example of VAC pump localization. In study 1, we administered a survey to assess clinic staff’s perceived comfort with and perceived usefulness of device localization and to identify any concerns related to its implementation. In study 2, we used an experimental design to test whether improving the layout of a privacy policy—featuring a guiding-question format as opposed to plain text—could enhance potential patients’ subjective understanding and whether a better understanding, in turn, could predict greater acceptance. Together, the 2 studies were designed to inform how digital health tools can be introduced in ways that are transparent, user-centered, and conducive to adoption. Rather than offering a broad theoretical advance, this research provides exploratory, context-specific evidence that links RTLS implementation concerns with consent and privacy-communication design in the concrete case of VAC pump localization.

## Methods

### Study 1

#### Participants

In a clinic in southern Germany, 2 wards were selected for recruitment, and volunteers were invited to participate. The final sample consisted of 38 clinic employees. Participants had a mean age of 33.18 (SD 11.92) years and reported working in the clinic for an average of 13.40 (SD 11.66) years. Most participants identified as female (n=27), followed by male (n=7), and a small number of participants preferred not to disclose their gender (n=4). Regarding professional roles, the majority worked in nursing (n=32), while the remaining participants reported working in other roles (n=3), in wound management (n=2), or as a physician (n=1).

#### Ethical Considerations

This study was conducted in accordance with the ethical principles of the Declaration of Helsinki and was approved by the Ethics Committee of the University of Bamberg, Germany (dossier/review number: 2023-11/23). All participants provided informed consent prior to participation. Participation was voluntary, and participants could discontinue at any time without penalty. Participants were informed about data handling during the consent process. Privacy and confidentiality were protected throughout the study; no directly identifying personal data are reported in this manuscript; study data were analyzed and reported in aggregate form only; and any contact information collected for the raffle was stored separately from the survey responses. As compensation, participants were offered the opportunity to win 1 of 10 gift vouchers worth €10 (US $11.70) each for the city of Bamberg, Germany.

#### Procedure and Measures

Before data collection, we obtained consent from the clinic’s work council. To minimize the disruption of routine clinical workflows, a printed questionnaire was distributed in 2 wards, where staff could complete it whenever their schedules permitted. The completed questionnaires were collected in sealed and nontransparent boxes to ensure anonymization. The survey remained available for a total of 29 days, from March to April 2024.

Attitudes toward the localization of VAC pumps were assessed with 2 self-developed items. Perceived comfort with localization was measured with the item: “How comfortable do you feel about the idea of localizing VAC pumps?” Responses were provided on an 11-point scale ranging from 0 (very uncomfortable) to 10 (very comfortable). Perceived usefulness was assessed with the item: “How helpful do you think the localization of VAC pumps would be?” Participants responded on the same 11-point scale ranging from 0 (not helpful at all) to 10 (very helpful). To minimize the burden during routine clinical work, these brief items were used as exploratory, descriptive indicators (not validated scales).

In addition, participants were asked an open-ended question: “Please describe any doubts or concerns you have about the localization of items or the project as a whole.” Responses were analyzed using qualitative content analysis. Thematic coding was conducted by the research team to group responses on the basis of content similarity.

Because of item-level missing responses, sample sizes vary slightly across study 1 outcomes; we report outcome-specific n values based on available cases for each item.

### Study 2

#### Preregistration

The analysis was preregistered on the Open Science Framework, and an anonymized version can be accessed by following the link in the references under files [16]. Any deviations from the preregistration are transparently reported and justified.

#### Participants

Participants were recruited online via the following three approaches: (1) snowball sampling, (2) calls posted in the University of Bamberg’s student forums, and (3) an announcement distributed through the university’s mailing list to individuals from the general population who had signed up to be informed about studies. As an incentive, participants were offered the opportunity to win one of forty €10 (US $11.70) gift vouchers for the city of Bamberg, Germany. Alternatively, psychology students could receive course credit for completing the study. A total of 498 individuals completed the online survey between November 2024 and February 2025. Of these, 308 identified as female, 182 as male, 6 as diverse, and 2 preferred not to disclose their gender. The mean age was 41.12 (SD 18.9) years. Regarding educational background, 38 participants reported basic education, 258 reported intermediate education, and 199 reported higher education, while 3 observations were missing. As this sample size exceeded the originally preregistered sample size of 310, we drew a random subsample of 155 female and 155 male participants for an additional analysis as a robustness check. Participants in the subsample had a mean age of 41.45 (SD 18.64) years. Regarding educational background, 23 participants reported basic education, 150 reported intermediate education, and 136 reported higher education. One participant did not provide information on their education.

#### Outlier Detection and Exclusion Criteria

To ensure data quality, careless responding was assessed using the relative speed index (RSI), which was calculated as the inverse of each participant’s total survey completion time relative to the group average. Participants with an RSI≥3, indicating exceptionally fast completion times, were to be excluded. However, no participants exceeded this threshold; thus, no exclusions were made based on the RSI.

#### Ethical Considerations

This study was conducted in accordance with the ethical principles of the Declaration of Helsinki and was approved by the Ethics Committee of the University of Bamberg, Germany (dossier/review number: 2025-05/39). All participants provided informed consent prior to participation. Participation was voluntary, and participants could discontinue at any time without penalty. Participants were informed about data handling during the consent process. Privacy and confidentiality were protected throughout the study; no directly identifying personal data are reported in this manuscript; study data were analyzed and reported in aggregate form only; and any contact information collected for the raffle was stored separately from the survey responses. As compensation, participants were offered the opportunity to win 1 of 40 gift vouchers worth €10 (US $11.70) each for the city of Bamberg, Germany. Psychology students could alternatively receive course credit for completing the study.

#### Measures

##### Subjective Understanding

Subjective understanding was assessed with 5 self-developed items rated on a 4-point Likert scale (1=strongly disagree, 4=strongly agree), including sample items such as “I feel that I understood the points listed.” We deliberately chose a 4-point scale without a neutral midpoint to discourage “safe” or noncommittal responses and to elicit clearly directional judgments about participants’ perceived understanding, while also keeping the response format cognitively simple in this applied setting. Responses were aggregated into a single composite score. Internal consistency was high (Cronbach *α*=0.86, 95% CI 0.837-0.877; McDonald *ω*=0.86, 95% CI 0.812-0.911). An exploratory factor analysis confirmed unidimensionality, with all factor loadings ranging from 0.68 to 0.81—well above the predefined threshold of 0.40.

##### Acceptance

Acceptance was measured with a single item: “How likely is it that you would agree to this privacy policy?” Participants responded on a slider scale ranging from 1 (representing 0%) to 101 (representing 100%). For the analyses, the scale was rescaled to range from 0 to 100.

### Design

Participants were randomly assigned to one of two types of information sheets. The two versions presented identical content; however, one version included guiding questions for each paragraph (eg, “What data are processed when the vacuum pumps are tracked?” and “Who has access to your data?”) to support comprehension. The information sheets (in German) were also uploaded to the Open Science Framework along with this preregistration. Participants were then asked to report their subjective understanding of the information sheet content and their acceptance. To test hypothesis 1—that including guiding questions in the layout of a privacy policy information sheet enhances subjective understanding as compared with a standard layout—we conducted a rank-based regression analysis. The dependent variable was subjective understanding, and the independent variable was the treatment condition (standard vs improved layout). Gender, age, education, and reading time were included as covariates to control for potential confounds.

To test hypothesis 2—that increased subjective understanding would lead to greater acceptance of the privacy policy—we conducted a rank-based regression analysis. Acceptance served as the dependent variable, and subjective understanding was included as the key predictor. Gender, age, and education were entered as covariates to control for potential confounding effects.

To further examine the relationship between layout, subjective understanding, and acceptance, we conducted an exploratory mediation analysis. We tested whether the effect of the layout condition (standard vs guiding questions) on acceptance was mediated by subjective understanding. Subjective understanding was modeled as the mediator and acceptance as the outcome. Both variables were analyzed in their original metric, with acceptance ranging from 0 to 100. We specified two linear regression models. In the mediator model, subjective understanding was regressed on layout condition, controlling for gender, age, education, and reading time. In the outcome model, acceptance was regressed on layout condition and subjective understanding, controlling for gender, age, and education (reading time was not included here to remain consistent with the main regression specification for acceptance). Indirect (average causal mediation effect [ACME]), direct (average direct effect [ADE]), and total effects were estimated using the mediation package in R with nonparametric bootstrap CIs based on 5000 simulations. Because this analysis relies on linear modeling and was not preregistered, we report it as exploratory and interpret it cautiously, using it to complement (but not replace) the rank-based regression analyses.

## Results

### Study 1

#### Perceived Comfort and Usefulness of VAC Pump Localization

**Figure 1.**
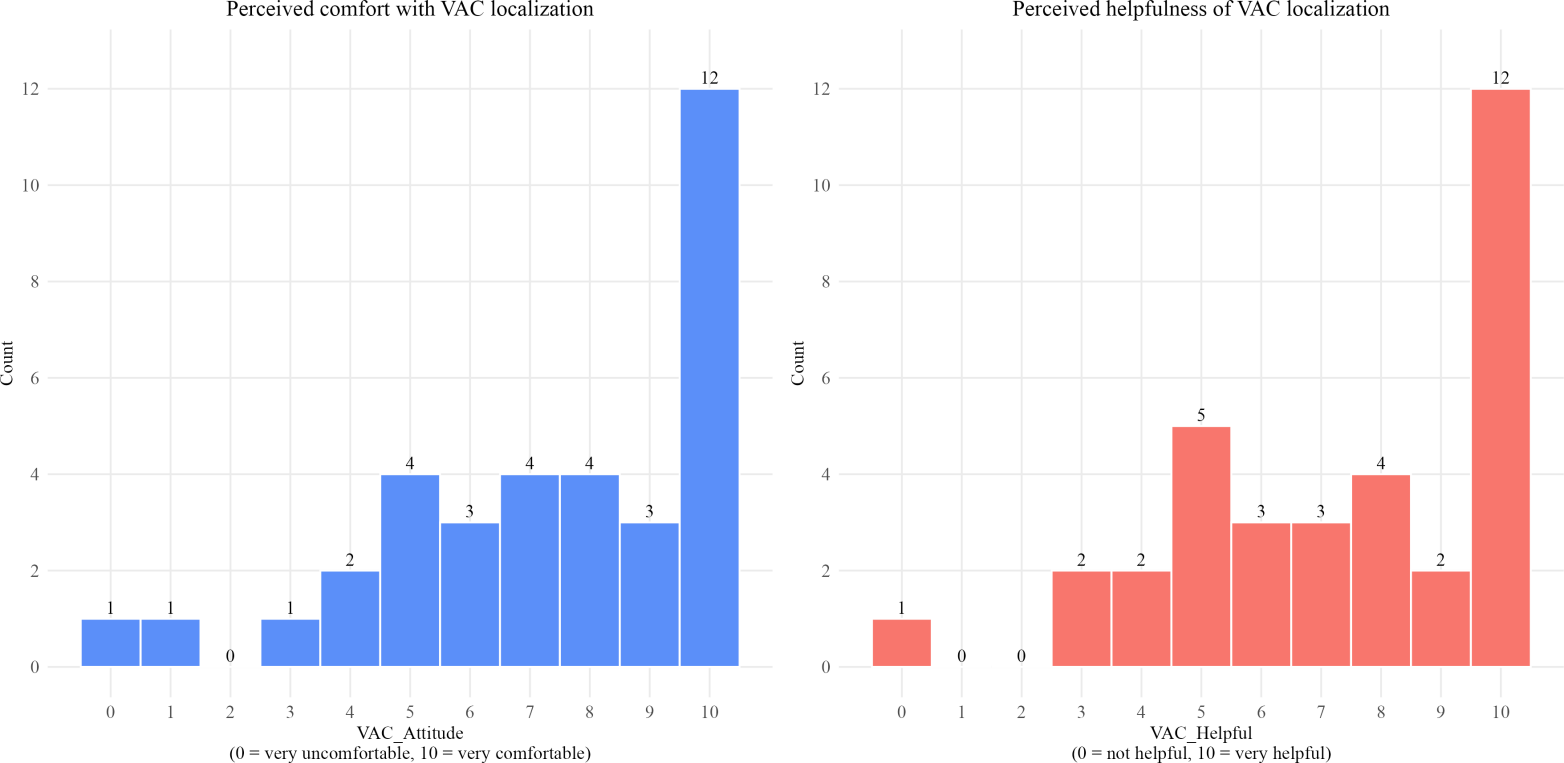
Staff perceptions of vacuum-assisted closure (VAC) pump localization. The histograms show the distributions of responses regarding perceived comfort (left) and perceived helpfulness (right) of localizing VAC pumps. Responses were given on a scale ranging from 0 to 10, with higher values indicating greater comfort or helpfulness.

Participants generally expressed positive attitudes toward the localization of VAC pumps. On a scale ranging from 0 (very uncomfortable) to 10 (very comfortable), the average level of comfort with the idea of localization was 7.34 (SD 2.75; n=35), see Figure 1.

Similarly, the perceived usefulness of localization was rated positively (mean 7.29, SD 2.69; n=34) on a scale ranging from 0 (not helpful at all) to 10 (very helpful). A visual inspection of the distributions indicated a slight positive skew, with the most frequent response being the highest value (10) for both items.

#### Staff Concerns Regarding Localization in Clinical Contexts

A total of 18 statements describing concerns about the localization of items in clinical practice were categorized across 5 themes. The most frequently mentioned concern was related to implementation feasibility (n=7), followed by technical doubts (n=3). Other participants questioned whether localization would be helpful, specifically for VAC pumps (n=2). Additional concerns included increased costs (n=2) and added workload for nursing staff (n=2).

### Study 2

#### Layout and Subjective Understanding

We hypothesized that including guiding questions in the layout of a privacy policy information sheet would enhance subjective understanding compared with a standard layout. A rank-based regression analysis was conducted with subjective understanding as the dependent variable; treatment condition (standard vs improved layout) as the predictor; and gender, age, education, and reading time as covariates. The primary analysis was conducted on the full sample, and the preregistered random subset consistent with the preregistered sample size was analyzed as a robustness check.

In the full sample, the analysis revealed a significant effect of treatment (*t*=2.57; *P*=.01), indicating higher subjective understanding in the improved layout condition compared with the standard layout (Table 1). Age was a significant positive predictor (*t*=3.91; *P*<.001), indicating that older participants reported a higher subjective understanding. No significant effects were found for gender, education, or reading time. The model explained 3.7% of the variance in subjective understanding (*R*²=0.037), and the reduction in dispersion test was significant (*P*=.006), indicating that the model provided a better fit than an intercept-only model. As a robustness check, the same analysis in the preregistered subset showed a marginal effect of treatment (*t*=1.93; *P*=.06) and a significant positive effect of age (*t*=1.99; *P*=.048); no significant effects were found for gender, education, or reading time. The model explained 2.5% of the variance in subjective understanding (*R*²=0.025), and the reduction in dispersion test was not significant (*P*=.27).

**Table 1.** Rank-based regression predicting subjective understanding^a^.

Predictor	Estimate (SE)	*t*	*P* value
Full sample (n=498)			
Intercept	3.1539 (0.1216)	25.93	<.001
Treatment	0.1273 (0.0495)	2.57	.01
Male	−0.0076 (0.0525)	−0.15	.89
Age	0.0054 (0.0014)	3.91	<.001
Education (intermediate)	−0.0011 (0.0967)	−0.01	.99
Education (higher)	0.0274 (0.0971)	0.28	.78
Reading time	−9×10^–6^ (7×10^–6^)	−1.28	.20
Preregistered random gender-balanced subsample (n=310)			
Intercept	3.3496 (0.1513)	22.14	<.001
Treatment	0.1185 (0.0615)	1.93	.06
Male	0.012 (0.0631)	0.19	.85
Age	0.0034 (0.0017)	1.99	.04
Education (intermediate)	−0.1176 (0.1223)	−0.96	.34
Education (higher)	−0.0869 (0.1217)	−0.71	.48
Reading time	−9×10^–6^ (7×10^–6^)	−1.25	.21

a Subjective understanding was predicted using rank-based regression. Treatment was coded as 0=standard and 1=improved layout. Reading time was measured in seconds. For the treatment comparison, subjective understanding in the full sample was a mean of 3.24 (SD 0.68; n=250) in the standard layout condition and a mean of 3.37 (SD 0.63; n=248) in the improved layout condition. In the preregistered gender-balanced subsample, the corresponding values were mean 3.26 (SD 0.64; n=160) and mean 3.40 (SD 0.61; n=150), respectively. Reported *t* values are coefficient test statistics from the rank-based regression model.

#### Subjective Understanding and Acceptance

To examine whether higher subjective understanding would support greater acceptance of the privacy policy (hypothesis 2), we conducted a rank-based regression with acceptance as the dependent variable and subjective understanding as the key predictor. Gender, age, and education were included as covariates. Again, the primary analysis was conducted on the full sample; the preregistered random subset consistent with the preregistered sample size was analyzed as a robustness check.

The full-sample model revealed a significant positive effect of subjective understanding on acceptance (Table 2; *t*=11.33; *P*<.001), indicating that participants who reported better understanding of the privacy policy also expressed a greater willingness to accept it. No significant effects were found for gender, age, or education (all *P*s>.32). The model explained approximately 13.8% of the variance in acceptance (robust *R²*=0.138), and the reduction in dispersion test was significant (*P*<.001), suggesting that the model provided a better fit than an intercept-only model. As a robustness check, the corresponding analysis in the preregistered subset likewise showed a significant positive effect of subjective understanding on acceptance (*t*=8.90; *P*<.001), whereas gender, age, and education were not significant. The model explained approximately 16% of the variance in acceptance (robust *R²*=0.16), and the reduction in dispersion test was significant (*P*<.001).

**Table 2.** Rank-based regression predicting acceptance^a^.

Predictor	Estimate (SE)	*t*	*P* value
Full sample			
Intercept	39.58 (5.03)	7.86	<.001
Understanding	13.32 (1.17)	11.33	<.001
Male	1.62 (1.63)	0.99	.32
Age	−0.01 (0.04)	−0.21	.84
Education (intermediate)	0.07 (2.99)	0.02	.98
Education (higher)	−0.46 (3.00)	−0.15	.88
Preregistered random gender-balanced subsample			
Intercept	40.36 (6.29)	6.42	<.001
** **Understanding	12.82 (1.44)	8.90	<.001
Male	0.57 (1.86)	0.31	.76
Age	0.05 (0.05)	1.06	.29
Education (intermediate)	−0.73 (3.58)	−0.20	.84
Education (higher)	0.49 (3.56)	0.14	.89

a Acceptance of the privacy policy was predicted using rank-based regression. Subjective understanding was entered as a continuous predictor.

#### Exploratory Mediation Analysis

In an exploratory step, we examined whether the effect of layout (standard vs guiding questions) on acceptance was mediated by subjective understanding. Using linear regression models with nonparametric bootstrapping, the indirect effect of layout on acceptance via subjective understanding (ACME) was small but statistically significant (ACME=2.26, 95% CI 0.64-4.32; *P*=.006) on a 0‐100 acceptance scale. The direct effect of layout on acceptance when controlling for subjective understanding (ADE) was not statistically significant (ADE=–1.48, 95% CI –6.68 to 3.66; *P*=.58), and the total effect of layout on acceptance was also nonsignificant (total effect=0.79, 95% CI –4.67 to 6.13; *P*=.78). Overall, this pattern of effects is consistent with an indirect pathway from layout to acceptance via subjective understanding, with no evidence of a remaining direct effect when subjective understanding is included.

## Discussion

### Study 1

Given the single-site convenience sample, our findings should be interpreted as context-specific and descriptive. The results of study 1 indicate that the staff appeared to be open to the idea of localizing VAC pumps to improve workflows and support them in their daily work. Their concerns were predominantly technical and infrastructural in nature, with no explicit articulation of privacy concerns about the localization of VAC pumps. This finding is encouraging, as privacy concerns have been identified as a potential hindrance to the successful implementation of such systems [9,10]. Nevertheless, it is essential that patients agree to the localization, as it involves their personal data. Ensuring ethical and informed consent necessitates not only patients’ acceptance of these systems but also their comprehension of the relevant information provided to them. Consequently, study 2 was designed to gain an understanding of the conditions under which potential patients can subjectively comprehend and accept information about VAC pump localization, with a particular emphasis on the impact of the guiding-question format compared with a plain text format.

### Study 2

Our results provide support for the hypothesis that incorporating guiding questions into privacy policy layouts enhances subjective understanding. This aligns with research showing that simplified and well-structured consent materials—using plain language, headings, and clearer layouts—improve comprehension [14,17-19]. While the variance explained in subjective understanding was modest, this is consistent with the fact that we manipulated only a single structural feature of the information sheet and did not account for other plausible determinants of perceived understanding, such as general cognitive ability and health literacy [20].

Interestingly, age emerged as a significant predictor in both analyses, with older participants reporting a greater subjective understanding regardless of condition. Several explanations could account for older participants reporting a greater subjective understanding of privacy policies. One possible explanation could be that older people have had more experience with health care. Older participants may have more extensive experience with health care settings and procedures, making them more confident in their understanding of health care–related documentation, including privacy policies. Another possible explanation could be their familiarity with traditional documentation. Older adults might be more accustomed to reading formal documents, such as consent forms and privacy policies, in their original formats, whereas younger participants might expect more interactive or digital presentations (eg, videos or app-like interfaces) [21]. An additional reason might be a self-reporting bias. The finding might reflect different calibrations in self-assessments rather than actual differences in understanding. Younger participants may be more critical of their own understanding, whereas older participants may overestimate their understanding in the sense of an illusion of explanatory depth [22]. Finally, the finding may have resulted from greater concerns about privacy among older adults [23]. Older participants might invest more attention in privacy-related content due to heightened privacy concerns, resulting in a better subjective understanding. This counterintuitive finding highlights the importance of testing information design approaches with diverse age groups rather than making assumptions about digital or information literacy based on age alone.

The data also supported our second hypothesis, demonstrating that higher subjective understanding significantly predicted greater acceptance. This relationship explained 13.8% of the variance in acceptance scores (robust *R*²=0.138), and the corresponding analysis in the preregistered subset yielded a comparable pattern (robust *R*²=0.16). This indicates that subjective understanding is a meaningful predictor of acceptance in this context. Conceptually, this aligns with work on data-intensive technologies—such as crowd-counting systems in smart cities—where better explanations and clearer communication are associated with higher trust and acceptance [24]. In our study, the exploratory mediation analysis yielded a small but statistically significant indirect effect of layout on acceptance via subjective understanding, while the total effect of layout on acceptance was not statistically significant. This pattern is compatible with the interpretation that the layout manipulation primarily affects acceptance via perceived understanding rather than exerting an additional direct influence; however, given the exploratory nature of the mediation analysis and the reliance on self-reported understanding, this inference should be treated cautiously. In general, our results are consistent with broader evidence that design-driven improvements in understanding are only one part of a larger “privacy calculus,” in which perceived benefits, trust, and risk perceptions jointly shape acceptance [25,26].

Building on study 1, which examined health care providers’ perceptions of VAC pump localization systems, in study 2, we shifted our focus to how structural elements of privacy policies affect user comprehension and acceptance. While study 1 highlighted clinical staff concerns, study 2 provided evidence that an improved layout can positively influence how patients perceive privacy information and, via subjective understanding, relate to acceptance judgments. At the same time, study 2 assessed subjective understanding rather than objective comprehension, and acceptance was measured as a single-item willingness judgment; thus, future studies should test whether similar layout manipulations improve performance-based comprehension and decision-relevant outcomes.

### General Discussion

#### Principal Findings

We examined attitudes toward RTLS-based equipment localization in health care, focusing on the example of VAC pump localization in 1 German clinic. Across 2 complementary studies, we explored how clinical staff perceive such technologies and how information sheet layout may influence patient understanding and acceptance.

Study 1 provided initial insights into the perspectives of clinic employees. Overall, participants expressed generally positive attitudes toward the idea of VAC pump localization, reporting high levels of comfort and perceived usefulness. These findings are consistent with prior work on RTLSs, which emphasizes perceived usefulness, workflow support, and safety gains as key drivers of staff acceptance [3,8]. At the same time, some employees voiced concerns about technical feasibility, implementation logistics, and the potential for an increased workload—issues that echo previous evaluations of RTLSs in hospital contexts, where organizational readiness and human-factors considerations often determine whether technically viable systems are used consistently in practice [3,10].

Study 2 experimentally tested whether the layout of a privacy policy influences subjective understanding and its association with acceptance. Controlling for gender, age, education, and reading time, a layout incorporating guiding questions significantly improved subjective understanding compared with a standard format. These results align with health-communication research, demonstrating that structured consent documents with headings, bullet points, and guiding prompts improve readability and comprehension, especially when baseline literacy is heterogeneous [13,14,17,19]. Among the covariates, only age showed a small positive association with subjective understanding, indicating that older participants felt slightly more confident in their understanding (as discussed above). Moreover, higher subjective understanding was associated with greater acceptance of the policy. These findings are consistent with prior evidence showing that structured consent materials—such as those using headings and guiding prompts—can improve comprehension and user engagement [14,15].

Taken together, the findings underscore the dual importance of content and context in the introduction of data-driven technologies in clinical environments. While technical feasibility and staff buy-in are essential for implementation success, the way information is presented also plays a critical role in shaping patients’ perceptions and willingness to engage with such systems. The studies provide complementary perspectives on the implementation of location-tracking technologies in health care settings. Study 1 revealed the nuanced attitudes and operational concerns of frontline staff toward VAC pump localization, and study 2 demonstrated how information sheet layout can impact patients’ understanding and acceptance of such systems. This complementarity is particularly valuable because even though the staff members in study 1 were very positive about the implementation of a localization system, patients might express concerns about their privacy, which study 2 then addressed by testing communication approaches that could mitigate these very concerns. Even though the material that participants in study 2 received was addressed to patients, it might be just as helpful for staff to understand the system. For example, the guiding-question format, which improved subjective comprehension among potential patients, might also be beneficial for addressing the operational concerns expressed by the clinic staff in study 1. Applying structured communication approaches in training sessions and staff information materials may help mitigate concerns about technical feasibility and implementation logistics. By improving staff members’ understanding of how the localization system works and what benefits it offers, their acceptance and willingness to use the system may increase. Greater staff acceptance, in turn, may enable more consistent use in everyday practice and thereby enhance efficiency in clinical workflows—for example, by reducing the time needed to locate equipment. In addition, confident and well-informed staff are likely to convey more trust to patients, thereby positively shaping patients’ perceptions and acceptance of the system. Thus, integrating the insights from both studies into a comprehensive communication strategy might simultaneously address staff concerns and patient privacy considerations, thereby facilitating more effective implementation of RTLS technologies in health care settings. Together, the 2 studies provide a more comprehensive picture than either study could have offered in isolation—combining a rich contextual understanding with experimental evidence on how to address key implementation barriers.

#### Limitations

Several limitations should be noted. The sample size in study 1 was relatively small and limited to a single clinical institution, thereby potentially reducing the generalizability of the findings. Given the voluntary nature of participation, staff who held stronger concerns may have been less likely to respond, which could have biased attitudes in a more positive direction. Moreover, attitudes toward VAC pump localization were assessed with 2 single self-developed items (comfort and perceived usefulness), which precludes an assessment of internal consistency and may have reduced measurement reliability. Because these items were not validated and we did not estimate a measurement model, the study 1 quantitative results should be interpreted as descriptive indicators in this specific setting rather than as precise or generalizable estimates of staff acceptance. We chose this brief format to keep the survey feasible in a busy clinical environment and obtain an initial indication of staff attitudes, but future studies should use multi-item scales and larger, multisite samples to provide more robust and generalizable evidence. Furthermore, the acceptance of object localization may depend on the specific type of item that is being tracked. While VAC pumps were seen as unproblematic, other objects—particularly those more closely associated with staff or patient movement—may raise greater concerns, including fears of increased surveillance or performance monitoring, which our data do not capture [9-11]. Generalization to other use cases should therefore be approached with caution.

In study 2, the measures relied on self-reported understanding and a single-item acceptance judgment rather than behavioral outcomes. Additionally, while the layout manipulation in study 2 targeted structural design, other features, such as language complexity and institutional trust, were not varied and may also influence understanding [13]. Moreover, participants’ subjective understanding was the sole focus of the assessment. Consequently, the study lacks the necessary data to determine whether participants’ comprehension was genuinely enhanced by the improved explanation or whether they merely perceived an improvement in understanding. Recent work in the context of location tracking in a different setting has shown that objective understanding, assessed with comprehension questions, can be more strongly related to acceptance—via increased project-specific trust—than subjective understanding alone [24]. Against this background, our reliance on subjective understanding may have attenuated the observed associations and could partly explain why the estimated indirect effect of layout on acceptance via understanding was small but statistically significant. Future studies should therefore combine subjective ratings with performance-based comprehension measures and, where possible, behavioral indicators of information use and technology acceptance.

#### Practical Implications

The results point to several actionable recommendations. First, incorporating clear, question-based formatting into privacy communications can support perceived understanding, as evidenced by study 2. Such formatting could be implemented by redesigning consent forms, digital interfaces, and educational materials throughout the health care facility.

Second, early engagement with clinic personnel—especially those responsible for operational logistics—is essential to address concerns and increase buy-in. This engagement should include structured opportunities for staff to provide feedback during all implementation phases, from initial planning to postdeployment evaluation. Implementation could include comprehensive training programs that not only focus on technical operation but also provide context about how the technology integrates with existing workflows and benefits patient care. Such information is especially important given that staff voiced concerns about potential increases in workload in study 1.

#### Future Directions

Future research could build on these findings in several ways. Longitudinal studies may help assess whether improved understanding leads to sustained trust and actual use over time. In addition, experimental variations in communication elements—such as video-based or interactive explanations—could shed more light on how to optimize privacy-related materials for clinical audiences [5]. Critically, future studies should directly compare subjective understanding with objective comprehension to determine whether layout- or format-driven changes translate into demonstrable knowledge gains. Building on these initial directions, future research should examine whether the age-related differences we observed in subjective understanding translate into actual differences in comprehension. Studies incorporating brief knowledge assessments (eg, multiple-choice or true or false items targeting key policy elements such as what data are collected, who can access them, and retention periods) alongside self-reported measures would provide valuable insights into this question, as earlier research found differences between objective and subjective understanding and their relationships with acceptance [24]. Where feasible, such work should also incorporate decision-relevant outcomes (eg, consent decisions or information-seeking behavior) rather than relying solely on self-report.

Additionally, comparative research across different health care institutions could help identify how contextual variables, such as organizational culture and existing technology infrastructure, influence staff attitudes toward location-tracking technologies. Such research would expand upon our single-institution findings and provide a more generalizable understanding of implementation factors.

#### Conclusion

As health care systems continue to adopt digital technologies, understanding how such innovations are communicated and perceived is becoming increasingly important. Within the specific context of VAC pump localization in one clinic and a hypothetical patient scenario, this research highlights 2 practical dimensions of successful implementation: the design of communication materials and the perspectives of clinical staff who are expected to engage with new systems. We showed that simple layout adjustments can significantly improve subjective understanding and are indirectly related to acceptance via perceived understanding. In our clinic sample, staff respondents generally welcome innovations such as equipment localization when they are presented transparently and aligned with clinical needs. At the same time, implementation must account for practical concerns on the ground. Together, these findings underscore the value of integrating human-centered design principles—in both communication and system planning—to ensure that digital innovations are not only functional but are also trusted and embraced by those who use them.

## Abbreviations

ACME: average causal mediation effect
ADE: average direct effect
RSI: relative speed index
RTLS: real-time location system
VAC: vacuum-assisted closure
